# Cumulative Exposure to Harsh Discipline at Home and School on Child Development: Evidence From Ghana

**DOI:** 10.1111/desc.70274

**Published:** 2026-08-18

**Authors:** Hang (Heather) Do, Yujie Fu, Richard Appiah, Esinam Ami Avornyo, Elisabetta Aurino, Sharon Wolf

**Affiliations:** ^1^ Graduate School of Education University of Pennsylvania Philadelphia Pennsylvania USA; ^2^ College of Health Sciences University of Ghana Accra Ghana; ^3^ Department of Psychology Northumbria University Newcastle upon Tyne UK; ^4^ Institute of Education University of Cape Coast Cape Coast Ghana; ^5^ School of Economics University of Barcelona Barcelona Spain

**Keywords:** academic skills, executive function, Ghana, harsh discipline, Sub‐Saharan Africa

## Abstract

Violence against children violates human rights and fundamentally challenges the global objectives outlined in the United Nations Sustainable Development Goals. Children may frequently experience harsh discipline in both homes and schools, two main settings for early learning and development, yet longitudinal evidence on the developmental consequences of exposure to harsh discipline across these two settings remains scarce in Sub‐Saharan Africa. Further, the interaction between harsh discipline at home and in school remains underexplored. Drawing on data from a longitudinal study in Ghana, we examine whether repeated exposure to harsh discipline at home and school across three years (which we define as “cumulative exposure”) among pre‐primary school children aged 5–8 years (*N* = 663; *M_age_* = 6.94 years). By controlling for baseline outcomes in a value‐added design, we estimated the robust associations between cumulative harsh discipline at home and at schools with higher‐order cognitive skills, namely academic and executive function. Cumulative harsh discipline at home significantly predicted lower academic skills (𝛽 = ‐0.045, *p* < 0.05) but not executive function, while cumulative harsh discipline at school did not statistically significantly predict any skills. Moderation analyses showed that children's experiences of harsh discipline at home were negatively associated with executive function under conditions of low levels of harsh discipline at school. Findings highlight the urgent need for integrated, contextually grounded interventions targeting home and school disciplinary practices to support early learning and development in Sub‐Saharan Africa.

## Summary

- Examines the associations between exposures to harsh discipline at home and school over 3 years and Ghanaian children's higher‐order cognitive skills.
- Harsh discipline at home across 3 years was associated with lower academic but not executive function skills.
- Harsh discipline at school across 3 years was not associated with child outcomes.
- Integrated efforts to reduce harsh discipline across home and school are needed.

## 1 Introduction

Violence against children fundamentally challenges the current Sustainable Development Goals (SDGs), particularly Target 16.2, which calls for “the end of all forms of violence against and torture of children” (United Nations 2016). Sub‐Saharan Africa is the world region with the highest rate of harsh discipline, though comparable rates in other regions may be underreported, Ghana ranks first globally, with 94% of children aged 1–14 exposed to some form of violence from adults, particularly 74.6% exposed to physical punishment and 86.7% to psychological aggression (Department of Children, Ministry of Gender, Children and Social Protection & UNICEF Ghana 2018; Egyir et al. 2026; UNICEF 2026). Despite this, research remains scarce on the consequences of such practices across systems, such as home and school, and over time on children's developmental trajectories.

As young children spend most of their time living, learning, and playing at home and school, understanding their exposure to harsh discipline across home and school over time and its associations with development and learning is key, particularly in countries where harsh discipline remains prevalent. While the occurrence of harsh discipline at home and school toward children is very high in Ghana (Egyir et al. 2026; UNICEF n.d), as in other Sub‐Saharan African settings, there is extremely limited knowledge about the developmental consequences of exposure to harsh discipline at home *and* school among children in these contexts simultaneously or their interaction over time (Evans et al. 2023). More importantly, among few existing studies examining the relations between harsh discipline from teachers and children's outcomes, they mostly focused on children under 5 years or adolescents, with little attention to those in primary schools (Evans et al. 2025). This creates a critical gap in understanding how exposure to harsh discipline in school affects children during middle childhood, a critical learning period when children aged 5–6 enter formal schooling and the developmental phase that lays the foundation for healthy adolescent development (Moilanen et al. 2010).

In this study, we define these exposures to physical and psychological aggression that children experience repeatedly over time as “cumulative harsh discipline,” in which “cumulative harsh discipline at home” refers to experience in the home over time and “cumulative harsh discipline at school” refers to experience in school over time. This study examines the associations and interactions between cumulative harsh discipline in two important contexts for childhood, home and school, and Ghanaian children's higher‐order cognitive skills in early primary school. We focus on two developmental domains, academic and executive function (EF) skills, as these play critical roles in children's human development trajectories.

### 1.1 The Ghanaian Context

Ghana is a lower‐middle‐income country in Sub‐Saharan Africa, where harsh discipline toward children is widely accepted as a strategy for correcting children's behavior (Kyei‐Arthur et al. 2024; Kyei‐Gyamfi et al. 2025). Although corporal punishment at school has been banned since 2017 (Abonyi and Salifu 2023), Ghanaian law continues to allow “reasonable” and “justifiable” punishment of children at home (End Corporal Punishment 2024), and both parents and teachers are calling for the reinstatement of corporal punishment in school (Ameamu 2025; Akyna and Manu 2024). Despite studies showing that non‐violent disciplines (such as calmly telling a child what they did was wrong) can support child development (Wolf and Suntheimer 2020; Dede Yildirim et al. 2020), many Ghanaian teachers and parents continue to perceive harsh discipline to be superior for managing children's behaviors (Abonyi and Salifu 2023; Quansah et al. 2023). While general evidence linking harsh discipline at home to children's learning and development is relatively well established, evidence in Ghana, especially on school‐based harsh discipline and children's cumulative exposure across home and school settings, remains extremely scarce.

Despite Ghana's relatively high pre‐primary and primary enrollment and completion rates (Ghana Ministry of Education 2018; World Bank 2020), a third of preschoolers still fail to meet basic developmental cognitive milestones (McCoy et al. 2016), and only one in five Ghanaian primary school‐aged children achieves minimum proficiency in math and reading (UNESCO 2022). By 2024, approximately 80% of Ghanaian children did not meet basic standards for numeracy and literacy skills by the end of their primary school (UNICEF 2024).

### 1.2 Home‐ and School‐Based Harsh Discipline and Child Development

Harsh discipline comprises physical punishment and psychological aggression. Physical punishment, including shaking, hitting, or slapping, is used to control the child's behavior through physical force rather than cause injury. It is usually divided into mild or severe punishment based on intensity (Straus 1994; UNICEF 2011). Psychological aggression involves emotionally harmful behaviors, such as shouting, screaming, or name‐calling, which is also considered violence against children (UNICEF 2011).

Research has shown that harsh discipline at home is consistently linked to impairments in children's EF and academic skills. A recent systematic review and multilevel meta‐analysis across 92 LMICs by Cuartas et al. (2025) found that physical punishment was significantly associated with impairment in academic outcomes (*d* = 0.24) and EF (*d* = 0.28). Psychological aggression was also associated with worse children's outcomes (*d* = 0.27) (Cuartas et al. 2025). A longitudinal cross‐cultural study in Bhutan, Cambodia, Ethiopia, and Rwanda found that children exposed to spanking scored significantly lower on literacy and numeracy, and evidence from Colombia showed that physical punishment was associated with lower receptive vocabulary among children and adolescents (Cuartas et al. 2020; Cuartas 2024). These findings warrant replication with samples in other contexts to understand children's exposure to harsh discipline at home and their subsequent academic outcomes.

Fewer studies have examined school‐based harsh discipline in relation to children's academic and EF skills. Although limited, existing evidence in West Africa reported that first‐grade children attending a punitive school, where corporal punishment was permitted, exhibited lower receptive vocabulary scores and poorer performance on self‐regulation, compared to their peers in a non‐punitive school (Talwar et al. 2011). In Jamaica and Gambia, primary school‐aged children who were exposed to school corporal punishment scored lower on math and reading, compared to children with no exposure (Baker‐Henningham et al. 2009; Gundersen and McKay 2019). One longitudinal study across four LMICs—Ethiopia, India, Peru, and Vietnam—demonstrated that corporal punishment in school at age 8 was associated with lower math and vocabulary scores at age 12, with heterogeneity by countries, providing evidence of longer‐term associations (Ogando Portela and Pells 2015).

While the negative relations between harsh discipline and child development outcomes have been separately found in home or school environments, the current literature is still limited in several important ways. Most research examines harsh discipline in either the home or the school, but limited research focuses on *both* contexts and their interaction. Second, while harsh discipline at school may differ in frequency, legitimacy, and public visibility compared to harsh discipline at home, existing studies are generally cross‐sectional (Devries et al. 2014; Maiti 2021), with extremely limited research on schools in the LMIC context (Evans et al. 2025). These existing gaps in the literature underscore the need for further investigation of children's concurrent exposure to harsh discipline across multiple contexts, particularly home *and* school settings.

### 1.3 Cumulative Exposure to Harsh Discipline and Child Development: A Conceptual Framework

The cumulative risk approach and empirical research using this framework have documented negative associations between the total number of adversity types an individual is exposed to and less favorable academic, cognitive, and social‐emotional outcomes across contexts (Evans et al. 2013). Yet it treats adversity exposure dichotomously and, as a result, does not fully capture the intensity and persistence of exposure to any type of adversity over time. Further examination into children's cumulative exposure to adversity across home and school—harsh discipline, in this case—is crucial for understanding how these experiences shape children's cognitive and learning abilities, particularly among children in low‐resourced contexts.

The interconnectedness of bioecological systems and the child's stress response systems are two hypothesized mechanisms that may explain the relations between harsh discipline at home and school on children's development. First, according to bioecological theory, children's development is shaped by proximal processes, defined as recurring interactions between the child and the people, activities, and symbols in the child's immediate environments, which unfold over time (Bronfenbrenner and Morris 2007). For young children, home and school are two especially important microsystems as they organize children's daily experiences of caregiving, behavioral regulation, learning, and social interactions. In addition, the interrelations between these core microsystems underscore the need to explore the relations between harsh discipline at home and school contexts in tandem instead of in isolation, where stressful experiences across interconnected microsystems may reinforce one another and may be associated with more unfavorable outcomes than in one setting (Margolin et al. 2010; McCoy 2013). These interconnected systems are embedded within a broader macrosystem of cultures and values that shape children's everyday experiences. In contexts where harsh discipline remains socially acceptable, such as Ghana, children's cumulative exposure to harsh discipline across both home and school may be intensified if cultural acceptance increases its practice across multiple settings, or lessened if children view it as an acceptable response from caregivers or teachers (e.g., Lansford et al. 2005).

Second, contemporary research in stress and neurobiology has shown that long‐term exposure to harsh discipline can overactivate the stress response systems and, therefore, is closely linked with cognitive development (Gershoff 2016). Toxic stress impairs the hippocampus’ ability to manage stress hormones in the body over time. This dysregulation further disrupts the growth of new brain cells needed for memory encoding and diminishes children's contextual learning and cognitive functioning (Rogosch et al. 2011; Shonkoff et al. 2012). These neurobiological effects have serious consequences on children's attention and learning abilities. Prolonged exposure to toxic stress and stressful interactions with parents, teachers at home and school can disproportionately and negatively affect the prefrontal cortex, which could inhibit children's higher‐order cognitive skills, such as emotional reactivity, executive function (EF), attention control, and learning via stress physiology (Miller and Cohen 2001; Cerqueira et al. 2007; Gunnar and Quevedo 2007; Evans and Schamberg 2009; Liston et al. 2011; Blair 2016). These skills are foundational for long‐term learning and development, and disruptions to EF and academic skills due to chronic stress may thus have cascading effects on later academic attainment and well‐being (Blair and Raver 2015).

### 1.4 Moderating Effects of Child Age and Sex

Evidence on how child sex and age shape children's exposure to harsh discipline across home and school settings within LMICs remains limited and mixed. While differences in developmental outcomes between boys and girls may be subtle and context‐dependent, gender socialization across cultural contexts may shape adults’ gender expectations and the differential treatment of children based on sex (Bem 1981; Lytton and Romney 1991; Bornstein et al. 2016; Deater‐Deckard and Lansford 2016; Endendijk et al. 2016). Adults holding stronger traditional gender role beliefs may be more likely to perceive boys as more active or “difficult” and that boys may be more likely to be exposed to harsh discipline than girls (Humphreys 2008; Baker‐Henningham et al. 2009; Hecker et al. 2014; Deater‐Deckard and Lansford 2016; Egyir et al. 2026). However, girls may experience more negative consequences of harsh discipline via shame or rumination, especially in contexts where their exposure to harsh discipline is relatively lower than boys (Smetana 2017; Iselin et al. 2022).

Similarly, although children in LMICs are exposed to harsh discipline at home as early as 2 years old (UNICEF 2014; Cuartas et al. 2019), findings on harsh discipline exposure by age are also mixed. Among Ghanaian urban preschoolers, younger children (aged 4–6) were more likely to experience physical punishment than older children (aged 7–11), and the negative association between punishment and numeracy skills was stronger among younger children (Wolf and Suntheimer 2020). In contrast, studies spanning broader age ranges suggest exposure may peak later, with physical and emotional violence at home being most prevalent among children ages 6–9 across LMICs (Egyir et al. 2026). Middle childhood is also a period when most children are enrolled in school, accentuating the risk of being exposed to further harsh discipline in the classroom. Whether these age differences extend in the relation between harsh discipline and academic outcomes, however, remains unclear due to a lack of longitudinal studies (Visser et al. 2022).

### 1.5 Present Study

This study addresses existing knowledge gaps by examining early primary school children's exposure to harsh discipline at home *and* school over 3 years in urban and peri‐urban Ghana. It contributes to the literature by providing evidence on cumulative experiences rather than isolated events to examine the trajectory of such exposures at both home and school in a Sub‐Saharan African context among children in middle childhood. Further, we explore its relations with children's higher‐order cognitive skills, particularly academic and EF skills, as well as the moderating effects of children's sex and age group on these associations. We address three research questions:

1. Does cumulative harsh discipline at home and school over 3 years predict Ghanaian children's academic and EF skills?
2. Do these relations differ between boys and girls and younger (5–7 years old) and older (8–10 years old) children?
3. Does cumulative harsh discipline at school separately moderate the association between cumulative harsh discipline at home and child outcomes?

We hypothesize that cumulative harsh discipline at home and school over 3 years will negatively predict children's higher‐order cognitive skills. In line with the cumulative risk framework, we also hypothesized that school‐based harsh discipline would moderate the relations between home‐based harsh discipline and child outcomes, such that children who were exposed to high levels of cumulative harsh discipline at *both* home and school would have the lowest scores. Given prior mixed findings in sex and age differences in the relations between children's exposures to harsh discipline at both home and school and their higher‐order cognitive skills, we treated the moderating role of child sex and age as exploratory.

## 2 Methods

### 2.1 Participants and Procedures

Data for this study were drawn from Waves 2 (June 2016), 3 (May 2017), and 4 (May 2018) of a school‐randomized trial that evaluated improvements in preschools in the Greater Accra region of Ghana (Wolf et al. 2019). There were 3407 children participating in 2016, who were randomly sampled from 444 classrooms and 240 schools across six districts in the Greater Accra region at baseline in 2015. This study was approved by the Institutional Review Boards of appropriate universities and organizations (Wolf et al. 2019). The teacher sample in the first two waves (2016 and 2017) included all KG teachers in selected schools from the study's baseline who were followed over 2 school years. Most schools had two KG teachers, but in cases with more, two were randomly sampled (one each from KG1 and KG2). For schools with only one teacher, that teacher was included. In 2018, as children had transitioned from pre‐primary to primary school, the child's current teacher was sampled.

Our analytic sample comprised 663 children (*M_age_* baseline = 5.21 years), which were children with available school violence data across all three waves and with complete outcome data in 2018. This was a reduced sample from the broader sample, due to substantial missing data on the harsh discipline at school variable in 2017 (approximately 62%), as classroom observations in this wave only followed teachers from the previous wave (2016). These were further limited given high teacher turnover rates in this sample (Oh et al. 2025). Sample attrition tests showed that children in the analytic sample (*n* = 663) had statistically significantly *higher* exposure to physical punishment at home and higher harsh discipline at school (*p* = 0.031 and *p* = 0.001, respectively) and statistically significantly *lower* average academic and executive function scores (*p* < 0.001) than the full sample of children collected in 2016 (*N* = 3407). Demographic characteristics between these samples were not statistically significantly different (see Table S1).

All child assessments were conducted in schools by trained Ghanaian data collectors proficient in English and local languages (Ga, Twi, Ewe, Dangme, and Hausa). Given the study's sensitive focus on disciplinary practices, enumerators received additional training on ethical data collection and safeguarding protocols. Upon arrival at schools, they identified quiet spaces for assessments and obtained verbal assent from children. Additionally, data collectors ensured that children were at ease before testing and assessed in the language that they were most comfortable with. Caregiver data were collected through 30‐min phone surveys at each wave. Primary caregivers, defined as the individuals primarily responsible for the child's education, provided data on household environments and sociodemographic characteristics. At baseline, 86.5% of respondents were biological parents, while 13.5% were other caregivers. They were, on average, 38.3 years old, and households were predominantly multilingual, with Twi (60.2%) and English (47.0%) being the most common languages spoken.

### 2.2 Measures

#### 2.2.1 Harsh Discipline at Home

Harsh discipline at home was assessed in 2016, 2017, and 2018 from caregiver surveys. The questions were adapted from the Parental Discipline Practices questionnaire in the *Multiple Indicator Cluster Survey* by UNICEF (2005), inquiring whether the caregiver or household member had any physical and psychological aggression toward the target child within the household over the past month. Physical punishment was assessed through six items, mentioning acts such as shaking, spanking, slapping, or beating the child in different body parts (e.g., “*Please tell me if you or someone else in your household spanked, hit, or slapped your child on the bottom with bare hand in the past month?*”). Similarly, psychological aggression was assessed through two items. An example item is: *“Please tell me if you or someone else in your household called child dumb or lazy in the past month?”*

For each wave, following previous work (Dede Yildirim and Roopnarine 2019; Wolf and Suntheimer et al. 2020; Lansford et al. 2022), responses to the eight items were summed to create a total score ranging from 0 to 8, with higher scores reflecting greater exposure to harsh discipline at home (𝛼 = 0.58–0.60 across waves). To establish cumulative exposure to harsh discipline at home, we first summed reported incidents of physical and psychological aggression within each wave (possible range: 0–8) and then averaged these sum scores across the three waves, yielding an average score ranging from 0 to 8 (0 = no harsh discipline reported in any wave; 8 = all eight forms of harsh discipline reported consistently across all three waves; *M *= 2.87, *SD* = 1.29).

#### 2.2.2 Harsh Discipline at School

Harsh discipline at school was measured in 2016, 2017, and 2018 based on the Classroom Management and Instructional Checklist created for the intervention study. A trained observer attended classroom sessions to record observation data about teachers’ engagement and classroom management activities toward children after observing the class for 30–60 min, focusing on whether each activity was present (= 1) and not present (= 0) in the classroom. All observers were Ghanaian, had at least a bachelor's degree, and attended a 5‐day training before classroom observations. Observations occurred once per classroom in each of the 3 years, and each observation was announced to teachers, where they were asked to provide consent for observation and classroom video‐taping. Inter‐rater reliability was assessed each wave, with 15% of videos independently coded by three raters (one of the raters being the live observer). Intraclass correlation coefficients (ICCs) were computed using the final scores to quantify the proportion of variance attributable to rater differences relative to agreement across raters. Variances shared across raters ranged from 71% to 80% across three waves, suggesting acceptable inter‐rater agreement (for further details of these assessments, see Wolf et al. 2019). The item that captures harsh discipline at school describes: *“Teacher threatens children with or used a cane on children at least once.”* We defined cumulative exposure to harsh discipline at school as the sum of a binary indicator of whether at least one incident of such violent behaviors was observed per classroom observation, based on 206 classroom observations conducted over three school years. A continuous scale ranging from 0 to 3 was created to reflect the cumulative exposure to harsh discipline at school across three waves (0 = no exposure to harsh discipline at school to 3 = exposure to harsh discipline at school across all waves; *M* = 0.39, *SD* = 0.66).

### 2.3 Child Outcomes

To assess the relations between cumulative harsh discipline at home and school and children's academic and EF outcomes, we drew on validated and culturally adapted assessment tools commonly used in low‐resource contexts. These included the *International Development and Learning Assessment* (IDELA; Pisani et al. 2018), *Early Grade Reading Assessment* (EGRA; RTI International 2016), and *Early Grade Math Assessment* (EGMA; RTI International 2014) for academic skills, and a range of EF tasks including cognitive flexibility, inhibitory control, and behavioral regulation.

#### 2.3.1 Academic Skills

*Literacy skills* in 2016 were directly assessed using the IDELA (Pisani et al. 2018), designed to be administered to children in low‐resource contexts and has been previously validated and utilized in Sub‐Saharan Africa (Halpin et al. 2019). To ensure children were assessed using their first language, we translated, back‐translated, and piloted into three common local languages in Ghana (Twi, Ewe, and Ga) to ensure semantic accuracy and cultural appropriateness. Adaptations were made in collaboration with local educators and field experts. Literacy skills questions comprised six subtasks, including print awareness, letter identification, first letter sounds, oral vocabulary, emergent writing, and oral comprehension. A composite score was created by averaging all subtasks, representing children's percent correct across subtasks (*M* = 0.55, *SD* = 0.19, range = 0–1; 𝛼 = 0.75). *Literacy* in 2018 was assessed using a combination of a phonological awareness subtask from the IDELA (Pisani et al. 2018) and four additional subtasks (letter‐sound identification, nonword decoding, expressive vocabulary, and listening comprehension) from the EGRA (RTI International 2016). The EGRA is a collection of subtasks designed to measure foundational reading skills in early primary grades and has been adapted to local languages and grade levels in Ghana. A composite literacy score was created by averaging all subtask scores to reflect the percent correct across all subtasks (*M* = 0.52, *SD *= 0.18, range = 0–1; 𝛼 = 0.75).

*Numeracy skills* in 2016 were measured using the IDELA (Pisani et al. 2018). It comprised 39 items divided into 10 subtasks: number identification, number discrimination, missing numbers, basic addition and subtraction, one‐to‐one correspondence, shape identification, sorting based on color and shape, size and length differentiation, pattern completion, and puzzle completion. We computed a composite score that reflects the child's overall percent correct by averaging the scores from all subtasks (*M* = 0.51, *SD* = 0.18, range = 0–1; 𝛼 = 0.79). *Numeracy* measures in 2018 were directly administered using the EGMA (RTI International 2014). This measure is also used in early primary grades and was adapted to local languages and grade levels in Ghana. The measure included six subtasks assessing a range of skills, including quantity discrimination (ability to compare quantities), missing numbers (completing number patterns), addition and subtraction, number identification, and word problems. Each subtask was scored binarily, with responses marked as correct or incorrect. A composite numeracy score was created by averaging the percentage of correct responses across each domain (*M* = 0.42, *SD* = 0.16, range = 0–1; 𝛼 = 0.82).

Finally, academic skills composite scores were created for analysis by averaging the literacy and numeracy skills (Wave 2: *M* = 0.53, *SD* = 0.18, range = 0–1; 𝛼 = 0.85; Wave 4: *M* = 0.46, *SD* = 0.16, range = 0–1; 𝛼 = 0.84). It was then standardized to have a mean of 0 and a standard deviation of 1 for analysis.

#### 2.3.2 Executive Function

*Executive Function* in Wave 2 (2016) was assessed using the IDELA (Pisani et al. 2018). It comprised three subtasks: short‐term memory, working memory (backward digit span), and inhibitory control (head‐toes task). Short‐term memory was assessed with a forward digit span task: the assessor read a sequence of digits aloud, and each child repeated them in the same order. In the head‐toes task, children were told to touch their toes whenever the assessor touched their head and vice versa. Each item on the measure was scored as correct or incorrect, and the composite score represents the percentage of items answered correctly (*M* = 0.55, *SD* = 0.19, range = 0–1; 𝛼 = 0.68).

*Executive Function* in 2018 was measured using three separate sub‐domains: cognitive flexibility, inhibitory control, and behavioral regulation. We selected these sub‐domains based on developmental relevance to early learning and self‐regulation, which are commonly disrupted under conditions of chronic stress (Blair 2016). *Cognitive flexibility* was assessed using the adapted version of the *Dimensional Change Card Sort‐Border* version (Zelazo 2006). The measure consisted of 12 items asking the child to sort cards by colors or shapes, and it has been used globally for young children. *Inhibitory control* was measured using the 21‐item adapted version of the Number Stroop Task (see Obradović et al. 2019 for information about measure adaptation). Assessor provided the child with a set of boxes with one to four repeated numbers (e.g., 1, 555, 22) and asked the child to report how many numbers were displayed in each box (i.e., child was not asked to answer what number was shown). *Behavioral regulation* was assessed using the adapted version of the Preschool Self‐Regulation Assessment‐Assessor Report (PSRA‐AS) (Smith‐Donald et al. 2007). This measure was developed for enumerators to assess children's emotional, attention, and behavioral self‐regulation during direct assessments. It was shortened and adapted for assessment in Zambia and other LMIC contexts (McCoy et al. 2015; Kim et al. 2020). Sample items include “*child pays attention during instructions and demonstrations*,*” “child remains in seat appropriately during test*,*”* and *“child modulates and regulates arousal level in self*.*”* Each item ranges from 1 to 4, with higher scores indicating better behavioral regulation.

Each domain score in 2018 was rescaled to 0–1 to reflect percent correct (*Cognitive flexibility*: *M* = 0.54, *SD* = 0.18, range = 0–1; 𝛼 = 0.79; *Inhibitory control*: *M* = 0.91, *SD* = 0.16, range = 0–1; 𝛼 = 0.93; *Behavioral regulation*: *M* = 0.92, *SD* = 0.09, range = 0–1; 𝛼 = 0.89), then all three domains were averaged to create a composite *Executive function* score (*M* = 0.79, *SD* = 0.10, range = 0–1; 𝛼 = 0.82). Finally, this composite score was standardized for analysis. Although different instruments were used across waves to ensure developmental appropriateness, there is some overlap in specific tasks across waves, such as inhibitory control, which was consistently measured across waves, providing a degree of measurement continuity as children grow while ensuring developmentally appropriate measures.

### 2.4 Covariates

Six covariates were included in the analyses, including child sex (1 = male), child age, child's school type (0 = public, 1 = private), caregiver education (0  =  none; 1  =  completed primary school; 2  =  completed junior high school; 3  = completed high school; 4  = more than high school but less than bachelor's; 5 = bachelor's degree and above), and teacher education (1 = junior high school; 2 = high school; 3 = more than high school but less than bachelor's; 4 = bachelor's degree; 5 = higher than bachelor's). Finally, we controlled for children's assessment outcomes at Wave 2 (2016).

### 2.5 Missing Data

While considerable effort was made to track all children and caregivers at each wave, the proportion of missingness in predictors and covariates across waves was minimal (<10%), mostly due to relocation or unreachable caregiver phone numbers. This missingness pattern is therefore considered missing at random (MAR). To handle missing data in remaining predictors and covariates, we employed multiple imputation by chained equations in R with 20 imputed datasets (Graham et al. 2007).

### 2.6 Analytic Plan

To examine the associations between cumulative exposure to harsh discipline at home and school and children's academic and EF skills, we ran a series of ordinary least squares regressions controlling for covariates and 2016 outcome variables. Such “value‐added” models estimate the association of earlier experiences and later developmental outcomes by accounting for baseline performance, thereby reducing bias from potential unobserved factors such as individual heterogeneity in ability, caregiver investments in children's learning, and other child‐ and household‐level characteristics associated with outcomes (Aurino et al. 2019). Separate models were estimated for each of the two outcomes.

Next, we estimated the moderating effects of child sex or age group at Wave 4 on these relations. To mitigate the effects of outlier values and ease analysis and interpretation, we top‐coded the age for three children above ten years old (two aged 11 and one aged 12) to 10 and divided them into two age groups: younger (5–7 years old) and older (8–10 years old). Moderation was tested by adding interaction terms between cumulative harsh discipline at home and child sex or age, and between cumulative harsh discipline at school and child sex or age, with sex and age being moderators in separate models. We then tested the moderating effects of cumulative harsh discipline at school on the associations between cumulative harsh discipline at home and two child outcomes using an interaction term between harsh discipline at home and school.

Finally, additional exploratory analyses tested the association between harsh discipline at home and school and each developmental sub‐domain (literacy, numeracy, cognitive flexibility, inhibitory control, and behavioral regulation) and the association between cumulative exposure to *severe* physical discipline at home (three items: adult hit child in the bottom or elsewhere in the body with hard objects; adult hit or slapped child on the face, head, or ears; and adult beat child repeatedly as hard as they could) predicting children's academic, EF, as well as each sub‐domain. Similar to cumulative harsh discipline at home, cumulative *severe* physical discipline at home was calculated by summing the total number of exposures within each wave and averaging across the three waves. Clustered standard errors were used in all models to adjust for the potential nesting of children within classrooms. Although the dataset spans multiple waves, children likely transitioned across classrooms, teachers, or schools as they progressed through primary schooling (e.g., kindergarten to primary school), making it methodologically challenging to account for clustering at the classroom or school level consistently across all waves. In addition, there were 432 classrooms being observed in 2016, compared to 347 classrooms observed in 2017, and 911 kindergarten and primary classrooms where children were spread out as they progressed through schooling. Thus, we clustered the standard errors for the classrooms that children attended in 2016, the first wave of data used in this study. All coefficients were standardized (*β*), allowing for interpretation in terms of standard deviation changes in outcomes per unit change in the predictor.

## 3 Results

Descriptive statistics and bivariate correlations for study variables are presented in Tables 1 and 2. Fifty percent of the children in the sample were male, and they were, on average, 7 years old in 2018. Overall, children experienced 2.9 harsh disciplinary practices at home over three years and 0.4 harsh disciplinary practices at school over three years. On average, caregivers in this sample completed junior/high school, while teachers completed high school but less than a bachelor's degree. Cumulative harsh discipline at home was not correlated with cumulative harsh discipline at school, suggesting the two experiences are independent, indicating children's exposure to harsh discipline in one setting does not necessarily suggest higher or lower exposure to harsh discipline in another setting.

**TABLE 1:** Descriptive statistics of study variables (*N* = 663).

Variable	M (SD)	Range
Child is male	0.50 (0.50)	0–1
Caregiver education at Wave 2	3.02 (1.45)	1–6
Teacher education at Wave 2	2.78 (0.75)	1–5
Child in private school at Wave 2	0.55 (0.50)	0–1
Child age	6.94 (1.03)	5–10
Harsh discipline at home		
Wave 2	3.12 (1.77)	0–8
Wave 3	2.88 (1.69)	0–8
Wave 4	2.60 (1.70)	0–8
Harsh discipline at school		
Wave 2	0.16 (0.36)	0–1
Wave 3	0.08 (0.27)	0–1
Wave 4	0.16 (0.36)	0–1
Cumulative harsh discipline at home (W2 to W4)	2.87 (1.29)	0–8
Cumulative harsh discipline at school (W2 to W4)	0.39 (0.66)	0–3
Wave 2 outcomes		
Academic skills	0.53 (0.18)	0.07–0.93
Executive function	0.55 (0.19)	0–0.89
Wave 4 outcomes		
Academic skills	0.46 (0.16)	0.06–0.83
Executive function	0.79 (0.10)	0.27–1

*Note*: M = Mean; SD = Standard deviation; W2 = Wave 2 (2016); W4 = Wave 4 (2018).

**TABLE 2:** Bivariate correlations among key study variables.

Variable	1.	2.	3.	4.	5.	6.	7.	8.	9.	10.
1. Child is female	—									
2. Child age	−0.08	—								
3. Child in private school	0.04	−0.21^***^	—							
4. Caregiver education	0.00	0.26^***^	−0.21^***^	—						
5. Teacher education	−0.03	−0.68^***^	0.08	−0.11^**^	—					
6. Cumulative harsh discipline at home	−0.08	−0.05	−0.04	−0.18^**^	0.01	—				
7. Cumulative harsh discipline at school	0.00	−0.13^***^	0.05	−0.11^**^	0.04	−0.03	—			
8. Academic Skills Wave 2 (2016)	0.07	0.29^***^	0.29^***^	0.14^***^	−0.15^***^	−0.09^*^	−0.01	—		
9. Executive Function Wave 2 (2016)	0.03	0.14^***^	0.23^***^	−0.01	−0.07	−0.07	−0.01	0.53^***^	—	
10. Academic Skills Wave 4 (2018)	0.02	0.40^***^	0.08	0.26^***^	−0.21^***^	−0.14^***^	−0.05	0.70^***^	0.44^***^	—
11. Executive Function Wave 4 (2018)	0.07	0.19^***^	0.03	0.10^*^	−0.13^**^	−0.10^*^	−0.03	0.36^***^	0.24^***^	0.49^***^

* *p* < 0.05.

** *p *< 0.01.

*** *p *< 0.001.

Tables S2, S3, and S4 display descriptive statistics on cumulative harsh discipline at home and school, stratified by children's sex, age group, and school types across three waves. For child sex and age group, although there were no significant differences, the tables indicate that boys tend to consistently experience more harsh discipline than girls. Younger children tend to experience more harsh discipline at home than older children in 2016 and 2017, but less in 2018, while they consistently experience less discipline than their older peers in all three waves at school. On the other hand, children in public schools were more likely to be exposed to physical punishment in 2016 and psychological aggression in 2017. Notably, they were significantly more likely to be exposed to harsh discipline at school than children in private schools in 2016 and 2018.

### 3.1 Associations Between Cumulative Harsh Discipline Predicting Children's Higher‐Order Cognitive Outcomes

Results from multivariate regression analyses are presented in Table 3. Cumulative harsh discipline at home significantly predicted lower academic scores (𝛽 = –0.046, *SE* = 0.022, *p* < 0.05), but not EF. In other words, each unit increase in cumulative harsh discipline at home was associated with a 0.046 SD decrease in academic outcomes.

**TABLE 3:** OLS result of cumulative harsh discipline at home and school predicting children's outcomes (*N* = 663).

	Academic skills	Executive function
	𝛽 (SE)	𝛽 (SE)
Cumulative harsh discipline at home	−0.046 (0.022)^*^	−0.038 (0.030)
Cumulative harsh discipline at school	−0.040 (0.040)	−0.034 (0.052)
Intercept	−1.399 (0.272)^***^	−0.892 (0.372)^*^
*R* ^2^	0.512	0.077

*Note*: All coefficients are standardized (𝛽). Clustered standard errors are shown in parentheses. Analyses controlled for full covariates (coefficients not shown).

Abbreviation: SE, standard error.

* *p* < 0.05.

** *p *< 0.01.

*** *p *< 0.001.

### 3.2 Moderation by Child Sex and Age Group

Table 4 displays the results of models testing the moderation effects of child sex on the associations between cumulative harsh discipline at home (first panel) and school (second panel) and children's outcomes. We found that there was a trend‐level statistically significant moderating effect of child sex on the association between cumulative harsh discipline at home and EF (𝛽 = 0.098, *SE* = 0.057, *p* < 0.10). There were no moderating effects of child sex on academic outcomes. Table 5 shows the results of moderation analyses of child age groups on the associations between cumulative harsh discipline at home (first panel) and school (second panel) and children's outcomes. There was no evidence for moderation by child age.

**TABLE 4:** Moderation analyses by child sex (*N* = 663).

	Academic skills	Executive function
	𝛽 (SE)	𝛽 (SE)
Child female	−0.020 (0.134)	−0.180 (0.178)
Cumulative harsh discipline at home	−0.040 (0.031)	−0.085 (0.045) +
Cumulative harsh discipline at home × Child female	−0.012 (0.044)	0.098 (0.057)+
Intercept	−1.490 (0.260)^***^	−0.658 (0.346) +
*R* ^2^	0.512	0.080
Child female	−0.095 (0.065)	0.142 (0.090)
Cumulative harsh discipline at school	−0.102 (0.059)+	0.009 (0.073)
Cumulative harsh discipline at school × Child female	0.133 (0.082)	−0.079 (0.103)
Intercept	−1.635 (0.233)^***^	−0.956 (0.343)^**^
*R* ^2^	0.511	0.075

*Note*: All coefficients are standardized (𝛽). Clustered standard errors are shown in parentheses. Analyses controlled for full covariates (coefficients not shown).

Abbreviation: SE, standard error.

* *p* < 0.05.

** *p *< 0.01.

*** *p *< 0.001.

*+ p* < 0.10.

**TABLE 5:** Moderation analyses by age group (*N* = 663).

	Academic skills	Executive function
	𝛽 (SE)	𝛽 (SE)
Child age	−0.157 (0.149)	−0.061 (0.199)
Cumulative harsh discipline at home	−0.046 (0.025)+	−0.043 (0.036)
Cumulative harsh discipline at home × Child age	0.006 (0.050)	0.015 (0.063)
Intercept	−1.742 (0.184)^***^	−0.393 (0.247)
*R* ^2^	0.513	0.072
Child age	−0.159 (0.081) +	−0.015 (0.060)
Cumulative harsh discipline at school	−0.047 (0.043)	0.006 (0.095)
Cumulative harsh discipline at school × Child age	0.045 (0.102)	−0.057 (0.117)
Intercept	−1.903 (0.154)	−0.550 (0.218)^*^
*R* ^2^	0.511	0.071

*Note*: All coefficients are standardized (𝛽). Clustered standard errors are shown in parentheses. Analyses controlled for full covariates (coefficients not shown).

Abbreviation: SE, standard error.

* *p* < 0.05.

** *p *< 0.01.

*** *p *< 0.001.

*+ p* < 0.10.

### 3.3 Moderation of Cumulative Harsh Discipline at Home by Cumulative Harsh Discipline at School

**FIGURE 1:**
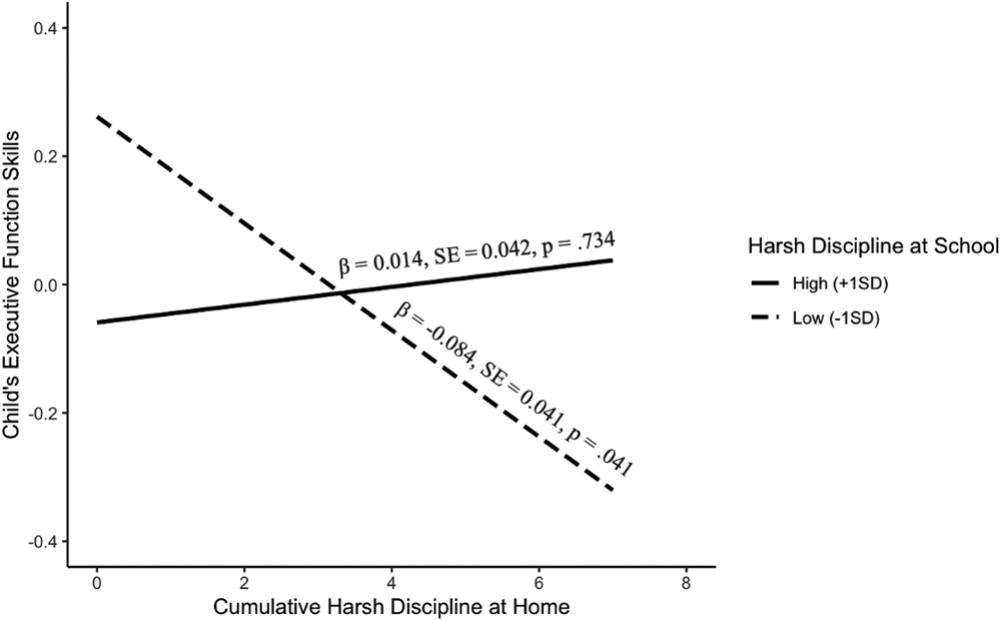
Moderating effects of cumulative harsh discipline at school on the relations between cumulative harsh discipline at home and children's executive function skills. The solid line indicates high levels (+1 SD) of cumulative harsh discipline at school; the dashed line indicates low levels (–1 SD) of cumulative harsh discipline at school. Under conditions of high levels of cumulative harsh discipline at school, there were no differences in EF scores by exposure to cumulative harsh discipline at home (𝛽 = 0.014, *SE* = 0.042, *p* = 0.734). For children experiencing lower levels of cumulative harsh discipline at school, higher levels of cumulative harsh discipline at home were significantly associated with lower EF scores (𝛽 = –0.084, *SE* = 0.041, *p* = 0.041). SE, standard error.

The final set of models examined how cumulative harsh discipline at school moderated the relations between cumulative harsh discipline at home and children's outcomes (see Table 6). There was a trend‐level statistically significant interaction term between cumulative harsh discipline at home and at school predicting lower EF scores (𝛽 = 0.074, *SE* = 0.043, *p* < 0.10). While only significant at the trend‐level, the descriptive pattern is worth noting: children who experienced low levels of harsh discipline at both home and school had the highest EF scores. Under conditions of high cumulative harsh discipline at school, EF scores did not differ by exposure to cumulative harsh discipline at home. For children experiencing lower levels of cumulative harsh discipline at school, higher level of cumulative harsh discipline at home was associated with lower EF scores (see Figure 1).

**TABLE 6:** Moderation analysis between cumulative harsh discipline at home and school predicting children's outcomes (*N* = 663).

	Academic skills	Executive function
	𝛽 (SE)	𝛽 (SE)
Cumulative harsh discipline at home	−0.060 (0.025)^*^	−0.064 (0.034) +
Cumulative harsh discipline at school	−0.157 (0.095) +	−0.243 (0.130) +
Cumulative harsh discipline at home × Cumulative harsh discipline at school	0.041 (0.032)	0.074 (0.043) +
Intercept	−1.356 (0.274)^***^	−0.815 (0.373)^*^
*R* ^2^	0.513	0.079

*Note*: All coefficients are standardized (𝛽). Clustered standard errors are shown in parentheses. Analyses controlled for full covariates (coefficients not shown).

Abbreviation: SE, standard error.

* *p* < 0.05.

** *p *< 0.01.

*** *p *< 0.001.

*+ p* < 0.10

### 3.4 Exploratory Analyses

We re‐ran our main models for each sub‐domain of children's outcomes (**Table** **S5**). Main effects showed that when fit separately (Models 1 and 2), cumulative harsh discipline at home predicted lower numeracy (𝛽 = –0.056, *SE* = 0.023, *p* < 0.05) and behavioral regulation scores (𝛽 = –0.081, *SE* = 0.032, *p* < 0.05), while cumulative harsh discipline at school was not a significant predictor for any domain. When modeled together (Model 3), cumulative harsh discipline at school predicted lower numeracy scores at the trend‐level (𝛽 = –0.068, *SE* = 0.041, *p* < 0.10). Finally, interaction results in Model 4 showed that there was a statistically significant interaction between cumulative harsh discipline at home and school predicting lower cognitive flexibility (𝛽 = –0.103, *SE* = 0.046, *p* < 0.05) but no other EF subdomains. Similar to results for composite EF scores, among children who experienced lower levels of harsh discipline at school, higher levels of harsh discipline at home were significantly associated with lower cognitive flexibility scores (see Figure S1; 𝛽 = –0.084, *SE* = 0.042, *p* = 0.044).

Cumulative *severe* physical punishment at home statistically significantly predicted children's both academic and EF skills (𝛽 = –0.165, *SE* = 0.057, *p* < 0.01 and 𝛽 = –0.191, *SE* = 0.076, *p* < 0.05), respectively. For sub‐domains, cumulative severe physical punishment at home statistically significantly predicted lower literacy (𝛽 = –0.147, *SE* = 0.065, *p* < 0.05), numeracy (𝛽 = –0.174, *SE* = 0.058, *p* < 0.01), and behavioral regulation skills (𝛽 = –0.231, *SE* = 0.093, *p* < 0.01), but not cognitive flexibility and inhibitory control.

## 4 Discussion

This study examined the associations between cumulative harsh discipline at home and school on Ghanaian children's academic and EF skills over 3 years. This is one of the first longitudinal studies in Sub‐Saharan Africa to examine the cumulative and interactive effects of harsh discipline across both home and school environments, addressing a critical evidence gap. The longitudinal design also allowed for controlling for lagged outcomes to strengthen the robustness of the associations examined. We found that harsh discipline at home, but not at school, was associated with lower academic outcomes. The association between cumulative harsh discipline at home and EF was stronger for boys than girls, and there was a trend‐level interaction between children's exposure to harsh discipline in both home and school predicting EF skills. These findings highlight the distinct and combined roles of home and school environments in shaping children's higher‐order cognitive skills.

Our key finding showed a statistically significant negative association between cumulative harsh discipline at home and children's outcomes, while harsh discipline at school did not, which may be driven by children's exposure to the severe forms of physical punishment at home. This finding highlights the home environment's crucial role in cognitive development (Hecker et al. 2016; Cuartas et al. 2025). Given the limited evidence from LMICs on the long‐term developmental impacts of harsh discipline, future studies should examine children's cumulative exposure over extended periods in regions, such as West Africa and broader Sub‐Saharan Africa, where social acceptance of harsh discipline remains high.

We did not find any associations between cumulative harsh discipline at school; however, this pattern should not be interpreted as an absence of harm but rather as indicative of limitations in the school‐based measure used in our analysis, due to multiple reasons. First, our dichotomous variable was relatively crude compared to the measure used to assess exposure to harsh discipline at home. This is a common limitation of measures used to capture school exposure, where researchers mostly rely on a single‐item questionnaire to teachers or students with Likert or yes/no responses (Devries et al. 2015; Gundersen and McKay 2019; Maiti 2021). Unlike previous studies, however, we did not rely on children's self‐reports of exposure to harsh discipline in the classroom or at school, but instead on direct classroom observations. This approach did not capture frequency, severity, or child‐specific exposure, as the observation applied to all children in the entire classroom, which likely undermines the variability of children's daily exposure to harsh discipline at school. Second, our prevalence estimates likely understate children's experiences in the classroom. Teachers may refrain from using harsh discipline when being observed (Fabbri et al. 2021), which may further introduce social desirability bias, despite harsh discipline being a common measure of behavioral management for children in this context. This speculation is corroborated by qualitative evidence showing harsh discipline remains common despite the 2017 ban by Ghana Educational Services (Danvers and Schley 2021). These issues could explain the low average cumulative exposure to school‐based harsh discipline observed in our data, and, via potential attenuation and social desirability biases, its nonsignificant association with children's outcomes.

Our exploration of moderation effects yielded some interesting trend‐level findings that should be interpreted with caution given their marginal statistical significance and small effect sizes. Nonetheless, we discuss them in order to help generate hypotheses to be tested in future research. First, the interaction between cumulative harsh discipline at home and school on EF outcomes highlighted a surprising trend and may suggest evidence for differential vulnerability based on exposure to harsh discipline across settings (Belsky and Pluess 2009). Specifically, children with high exposure to cumulative harsh discipline at both home and school may be more habituated to stress, which may result in a vulnerable prefrontal cortex and reduce the responsiveness of their EF systems (Grissom and Bhatnagar 2009; Blair 2016). This suggests a potential numbing effect at high levels of cumulative harsh discipline at both home and school. Additional research with larger samples and more comprehensive measures of harsh discipline at school is needed, both within Ghana and other countries within the LMIC context.

Next, we find suggestive evidence that child sex moderates the association between cumulative harsh discipline at home and EF, showing a tendency for stronger associations between exposure to higher levels of harsh discipline at home across three years and EF (but not academic skills) among boys. These findings suggest that boys’ actions may be considered “misbehavior” that warrants harsher disciplinary actions at home and school more so than for girls (Deater‐Deckard and Lansford 2016), which, in turn, may lead to more externalizing behaviors and provoke further punitive responses among boys. This suggestive finding invites further research to continue investigating the differential effects of cumulative harsh discipline across settings among boys and girls.

When we explored follow‐up associations by sub‐domain within academic and EF and of harsh discipline, cumulative harsh discipline was associated with numeracy but not literacy skills, consistent with the dimensional model of adversity whereby threat impacts associative learning while deprivation affects language acquisition (Wolf et al. 2019; Aurino et al. 2020). In contrast, severe physical punishment was associated with both outcomes, suggesting that extreme threat may broadly undermine learning (e.g., Sheridan et al. 2012). However, findings on how stress differentially affects academic outcomes remain inconsistent and warrant further investigation. Cumulative harsh discipline and severe physical punishment were also associated with lower behavioral regulation scores but not with any other EF subcomponents. This may reflect that children exposed to harsh discipline become more hypervigilant to hostile cues and display more disruptive behavior (Gershoff 2002). These patterns may reflect differing developmental trajectories or neurobiological sensitivities, though additional work is needed to confirm these domain‐specific effects. Finally, we found a significant interaction between cumulative harsh discipline and cognitive flexibility but not inhibitory control, which may reflect differences in sensitive periods across EF domains (Anderson 2002). Yet, these mechanisms have been mostly documented based on data from high‐income contexts, while the developmental timing of these domains might differ for children in LMICs due to variability in cultures, contexts, and access to resources (e.g., Modrek and Wolf 2024). Thus, future work should consider and investigate whether these patterns are similar or different within other countries in other samples in Ghana, Sub‐Saharan Africa, or other LMICs.

### 4.1 Limitations and Future Directions

The results of this study should be interpreted with consideration of its limitations. First, as mentioned, our measure of school harsh discipline was not comprehensive, as it contained only one binary item based on one classroom observation period at each wave, limiting a fuller understanding of children's daily experiences in their schools and classrooms. The mismatch in exposure timeframes between measures at home, which was assessed using caregiver reports of harsh disciplinary behaviors in the past month. On the other hand, school exposure only captures a single 30–60‐minute classroom observation in each year, potentially captures the lowest bound of actual prevalence of harsh discipline children were exposed to in the classroom and limits direct comparison across settings. These limitations may lead to an underestimate of the associations between harsh discipline at school and child outcomes. Importantly, data availability on harsh discipline at school globally, especially in the LMIC context, is extremely limited (Evans et al. 2023), and data that include measures of discipline at home and school contemporaneously are even scarcer. Future research should develop more nuanced and detailed measures of school‐based harsh discipline by directly asking children about their experiences and the intensity of the occasion, complemented by more frequent and extended classroom observations.

Second, the questionnaire items on harsh discipline at home only measured incidents within the past month and did not capture the frequency of harsh disciplinary practices across time, which might not have fully captured children's experience and exposure to harsh discipline at home. Additionally, although harsh discipline is widely practiced and culturally normative in Ghana, where physical punishment has been documented as a broadly accepted child‐rearing strategy (Kyei‐Gyamfi 2011; Twum‐Danso Imoh 2013), social desirability bias may be of concern, in which caregivers may underreport the use of their harsh discipline. More comprehensive measures, including items that cover the intensity of the incidents and are directly administered to children and other adults within the family or community, may be needed to ensure accuracy in measuring children's exposure to harsh discipline at home.

Third, the results presented are associational and do not shed light on causal relations between cumulative exposure to harsh discipline across home and school settings. Regardless, our study still provided robust estimates on the relations between harsh discipline at home and at school and child outcomes by using clustered standard errors and employing the “value‐added” models. Future research should employ causal inference methods and more comprehensively account for potential confounders to provide a more nuanced understanding of how cumulative harsh discipline across multiple settings shapes child development in Ghana and the broader Sub‐Saharan African context.

Finally, since participants were concentrated in one region, external validity is limited, even across other settings in Ghana. Despite this, Greater Accra is a region that reflects diverse school types and household profiles, offering insights that may be indicative of broader trends in urban and peri‐urban settings in Ghana. Importantly, missing data for harsh discipline at school in 2017 led to the exclusion of a large portion of our sample. The analytic sample had higher physical punishment at home, harsher discipline at school, and lower academic and EF scores than the full 2016 sample, suggesting that the analytic sample disproportionately represented children facing greater disadvantages, a pattern that most plausibly might have biased estimated effects toward attenuation.

Despite these limitations, the study substantially contributes to the literature by combining unique, multi‐modal forms of data to understand children's experiences of harsh discipline across both home and classroom settings. This is particularly important in contexts such as Ghana, where harsh discipline by adults remains a common and socially accepted approach to children's behavior management at home (Kyei‐Gyamfi 2011; Twum‐Danso Imoh 2013), and where a large proportion of children still fail to meet foundational cognitive milestones (Ghana Ministry of Education, 2018). Still, future research should consider including representative samples of schools, households, and children with careful consideration of attrition rates and missing data in longitudinal studies.

### 4.2 Conclusion and Implications for Policy and Practice

This study is among the first longitudinal studies in Sub‐Saharan Africa to explore how cumulative exposure to harsh discipline at home and in school predicts Ghanaian school children's academic and EF outcomes, and provides critical and novel evidence to global efforts to end violence against children, supporting the targets and indicators of the United Nations’ SDG Target 16.2 on ending all forms of violence against children. Despite a formal ban on harsh discipline in Ghanaian schools, it remains widely practiced and its reinstatement is actively debated; there is much to be learned about how it affects children's development and school experiences. Given that harsh discipline in Ghana, as in many LMICs, is pervasive and entrenched, additional efforts should be made at home and in schools to help mitigate the use of harsh discipline among children. Effective family interventions must facilitate discourse around non‐violent discipline at home, acknowledging cultural norms while promoting positive, strengths‐based parenting approaches through positive elements of the Ghanaian culture (e.g., children are gifts to society) (Department of Children, Ministry of Gender, Children and Social Protection & UNICEF Ghana 2018). To uphold the national policy of banning corporal punishment, schools should provide sufficient information to teachers about the harms of harsh discipline and the benefits of non‐violent discipline. Future policy and practice should provide culturally relevant caregiving resources for parents and teachers, encourage non‐violent disciplinary practices across settings, and guide international efforts to create safe, nurturing home and classroom environments for children. Finally, future research should build on this study by examining how positive discipline interventions predict longitudinal child outcomes and developing more nuanced measures to help capture harsh discipline in school settings.

## Author Contributions

**Hang (Heather) Do**: conceptualization, investigation, writing – original draft, methodology, visualization, writing – review and editing, software, formal analysis, data curation. **Yujie Fu**: writing – original draft, software, formal analysis, writing – review and editing. **Richard Appiah**: writing – original draft, writing – review and editing. **Esinam Ami Avornyo**: writing – original draft, writing – review and editing. **Elisabetta Aurino**: investigation, funding acquisition, writing – original draft, validation, writing – review and editing, project administration, supervision, resources. Sharon Wolfd: investigation, funding acquisition, writing – original draft, methodology, validation, writing – review and editing, project administration, data curation, supervision, resources.

## Funding

E.A. acknowledges the support of the Jacobs Foundation [grant number 2021‐1417‐00]; Spanish Ministry of Science, Innovation, and Universities [grant number PID2023‐150712NA‐I00, MICIU/AEI/10.13039/501100011033 and FEDER, UE, and MICIU/AEI/10.13039/501100011033 and European Union NextGenerationEU/PRTR]; and European Research Council funded by the European Union [grant number 101041741]. Views and opinions expressed are, however, those of the authors only and do not necessarily reflect those of the European Union, the European Research Council Executive Agency, or any of the other funders. Neither the European Union nor the granting authority can be held responsible for them.

## Conflicts of Interest

The authors declare no conflicts of interest.

## Supporting information




**Supporting information**: desc70274‐supp‐0001‐SuppMat.docx

## Data Availability

The first two waves of data (2016 and 2017) used in this study are openly available in the World Bank Data Catalogue at https://datacatalog.worldbank.org/dataset/ghana‐quality‐preschool‐impact‐evaluation‐2017, reference number [GHA_2017_QPIE‐EL_v01_M]. Data from the final wave (2018) can be obtained upon reasonable request from S.W. and E.A.
