# Supplementary material for: Cumulative Exposure to Harsh Discipline at Home and School on Child Development: Evidence From Ghana
Source: Dev Sci. 2026 Aug 18;29(5):e70274. doi: 10.1111/desc.70274 (PMC13487363; doi:10.1111/desc.70274)
Supplement: Supplementary file 1 [file DESC-29-e70274-s001.docx]

**Supplementary Table S1**

*Sample attrition of children in QP4G Wave 2 (2016) sample (N = 3,407) and analytic sample (N = 663)*

| **QP4G Wave 2 characteristics** | **N** | **In QP4G Wave 2**  **(N = 3,407)**  **M (SD)** | **N** | **Analytic sample**  **(N = 663)**  **M (SD)** | **Pairwise t-test mean difference** | **p-value** |
| --- | --- | --- | --- | --- | --- | --- |
|  |  |  |  |  |  |  |
| Caregiver is married | 2488 | 0.790 | 632 | 0.821 | -0.031 | 0.085 |
|  |  | (0.408) |  | (0.383) |  |  |
| Caregiver is female | 2651 | 0.522 | 647 | 0.499 | 0.023 | 0.294 |
|  |  | (0.500) |  | (0.500) |  |  |
| Household wealth | 2473 | 60.611 | 629 | 61.448 | -0.837 | 0.167 |
|  |  | (13.585) |  | (13.513) |  |  |
| Caregiver education | 2488 | 2.982 | 631 | 3.019 | -0.037 | 0.574 |
|  |  | (1.485) |  | (1.447) |  |  |
| Child is male | 2975 | 0.506 | 663 | 0.496 | 0.010 | 0.642 |
|  |  | (0.500) |  | (0.500) |  |  |
| Child in private school | 3407 | 0.532 | 663 | 0.554 | -0.022 | 0.299 |
|  |  | (0.499) |  | (0.498) |  |  |
| Physical punishment at home | 2335 | 1.913 | 617 | 2.057 | -0.144 | **0.031** |
|  |  | (1.475) |  | (1.458) |  |  |
| Psychological aggression at home | 2335 | 1.051 | 617 | 1.065 | -0.014 | 0.619 |
|  |  | (0.622) |  | (0.623) |  |  |
| Harsh discipline at home | 2335 | 2.978 | 617 | 3.124 | -0.146 | 0.072 |
|  |  | 1.802 |  | (1.766) |  |  |
| Harsh discipline at school | 3249 | 0.110 | 657 | 0.155 | -0.045 | **0.001** |
|  |  | (0.312) |  | (0.362) |  |  |
| Academic outcome | 3407 | 0.595 | 653 | 0.531 | 0.064 | **<.001** |
|  |  | (0.182) |  | (0.178) |  |  |
| Executive function | 3407 | 0.587 | 653 | 0.546 | 0.041 | **<.001** |
|  |  | (0.184) |  | (0.196) |  |  |

| **Supplementary Table S2**  *Descriptive statistics of harsh discipline at home and school by waves, stratified by child sex (N = 663)* | | | | | | | | | | | | |
| --- | --- | --- | --- | --- | --- | --- | --- | --- | --- | --- | --- | --- |
|  | **2016** | | | | **2017** | | | | **2018** | | | |
|  | ***Average*** | ***Boy*** | ***Girl*** | ***p*** | ***Average*** | ***Boy*** | ***Girl*** | ***p*** | ***Average*** | ***Boy*** | ***Girl*** | ***p*** |
| ***Physical punishment*** |  |  |  |  |  |  |  |  |  |  |  |  |
| Adult shook child | 27.5% | 26.7% | 28.1% | 0.736 | 22.0% | 22.5% | 21.6% | 0.644 | 24.9% | 25.5% | 24.3% | 0.753 |
| Adult spanked, hit, or slapped child in the bottom with bare hands | 48.6% | 51.7% | 45.5% | 0.079 | 50.4% | 51.1% | 49.7% | 0.567 | 38.0% | 39.5% | 36.5% | 0.456 |
| Adult hit child in the bottom or elsewhere in the body with hard objects | 44.6% | 47.1% | 42.2% | 0.203 | 44.6% | 45.6% | 43.7% | 0.525 | 29.6% | 30.4% | 28.7% | 0.721 |
| Adult hit or slapped child on the face, head, or ears | 10.1% | 12.5% | 7.8% | 0.060 | 7.8% | 8.5% | 7.2% | 0.558 | 6.6% | 9.4% | 3.9% | **0.006** |
| Adult hit or slapped child on the hand, arm or leg | 49.9% | 51.1% | 48.8% | 0.502 | 44.0% | 42.9% | 45.2% | 0.807 | 42.5% | 42.9% | 42.2% | 0.849 |
| Adult beat child repeatedly as hard as they could | 9.0% | 9.7% | 8.4% | 0.684 | 6.0% | 6.7% | 5.4% | 0.534 | 6.9% | 7.3% | 6.6% | 0.837 |
| *Total physical punishment items [M (SD)]* | 2.06 (1.46) | 2.17 (1.44) | 1.94 (1.47) | **0.049** | 1.81 (1.37) | 1.85 (1.40) | 1.77 (1.33) | 0.478 | 1.50 (1.35) | 1.57 (1.44) | 1.43 (1.26) | 0.198 |
|  |  |  |  |  |  |  |  |  |  |  |  |  |
| ***Psychological aggression*** |  |  |  |  |  |  |  |  |  |  |  |  |
| Adult shouted, yelled at or screamed at child | 76.2% | 76.6% | 75.7% | 0.998 | 78.7% | 78.7% | 78.7% | 1.000 | 78.4% | 80.5% | 76.3% | 0.311 |
| Adult called child dumb, lazy, or similar | 23.7% | 23.1% | 24.3% | 0.819 | 25.5% | 26.4% | 24.6% | 0.537 | 30.8% | 30.7% | 30.8% | 1.000 |
| *Total psychological aggression items [M (SD)]* | 1.06 (0.62) | 1.06 (0.60) | 1.07  (0.64) | 0.806 | 1.07 (0.62) | 1.09 (0.64) | 1.05 (0.60) | 0.417 | 1.10 (0.66) | 1.12 (0.64) | 1.08 (0.69) | 0.507 |
| *Total harsh discipline (physical & psychological items combined) [M (SD)]* | 3.17  (1.77) | 3.22  (1.72) | 3.03  (1.81) | 0.173 | 2.88 (1.69) | 2.94 (1.76) | 2.82 (1.62) | 0.409 | 2.60 (1.70) | 2.68 (1.77) | 2.52 (1.63) | 0.227 |
|  |  |  |  |  |  |  |  |  |  |  |  |  |
| ***Harsh Discipline at school:*** teacher threatened or hit child with a cane | 15.4% | 15.5% | 15.3% | 0.993 | 8.1% | 8.8% | 7.5% | 0.629 | 15.7% | 14.6% | 16.8% | 0.507 |

| **Supplementary Table S3**  *Descriptive statistics of harsh discipline at home and school by waves, stratified by child age group (younger; 5-7 years vs. Older; 8-10 years) in 2018*  *(N = 663)* | | | | | | | | | | | | |
| --- | --- | --- | --- | --- | --- | --- | --- | --- | --- | --- | --- | --- |
|  | **2016** | | | | **2017** | | | | **2018** | | | |
|  | ***Average*** | ***Younger*** | ***Older*** | ***p*** | ***Average*** | ***Younger*** | ***Older*** | ***p*** | ***Average*** | ***Younger*** | ***Older*** | ***p*** |
| ***Physical punishment*** |  |  |  |  |  |  |  |  |  |  |  |  |
| Adult shook child | 27.5% | 28.9% | 22.8% | 0.188 | 22.0% | 22.8% | 19.8% | 0.538 | 24.9% | 25.0% | 24.7% | 1.000 |
| Adult spanked, hit, or slapped child in the bottom with bare hands | 48.6% | 52.3% | 37.0% | **<.001** | 50.4% | 50.7% | 49.4% | 0.980 | 38.0% | 37.9% | 38.3% | 0.967 |
| Adult hit child in the bottom or elsewhere in the body with hard objects | 44.6% | 42.7% | 50.6% | **0.046** | 44.6% | 44.3% | 45.7% | 0.789 | 29.6% | 28.3% | 33.3% | 0.259 |
| Adult hit or slapped child on the face, head, or ears | 10.1% | 9.0% | 13.6% | 0.104 | 7.8% | 7.6% | 8.6% | 0.776 | 6.6% | 6.4% | 7.4% | 0.776 |
| Adult hit or slapped child on the hand, arm or leg | 49.9% | 51.3% | 45.7% | 0.251 | 44.0% | 45.1% | 40.7% | 0.399 | 42.5% | 42.7% | 42.0% | 0.957 |
| Adult beat child repeatedly as hard as they could | 9.0% | 8.8% | 9.9% | 0.708 | 6.0% | 6.2% | 5.6% | 0.957 | 6.9% | 6.8% | 7.4% | 0.926 |
| *Total physical punishment items [M (SD)]* | 2.06 (1.46) | 2.08  (1.44) | 1.97  (1.53) | 0.427 | 1.81 (1.37) | 1.82 (1.37) | 1.76 (1.36) | 0.637 | 1.50 (1.35) | 1.48  (1.30) | 1.55 (1.50) | 0.621 |
|  |  |  |  |  |  |  |  |  |  |  |  |  |
| ***Psychological aggression*** |  |  |  |  |  |  |  |  |  |  |  |  |
| Adult shouted, yelled at or screamed at child | 76.2% | 78.2% | 69.8% | **0.047** | 78.7% | 80.2% | 74.1% | 0.195 | 78.4% | 79.2% | 75.9% | 0.511 |
| Adult called child dumb, lazy, or similar | 23.7% | 22.2% | 28.4% | 0.082 | 25.5% | 25.9% | 24.1% | 0.795 | 30.8% | 29.5% | 34.6% | 0.275 |
| *Total psychological aggression items [M (SD)]* | 1.06 (0.62) | 1.07 (0.61) | 1.06 (0.66) | 0.928 | 1.07 (0.62) | 1.09 (0.61) | 1.03 (0.64) | 0.293 | 1.10 (0.66) | 1.09  (0.65) | 1.12 (0.70) | 0.664 |
| *Total harsh discipline (physical & psychological items combined) [M (SD)]* | 3.17  (1.77) | 3.15 (1.71) | 3.06  (1.93) | 0.600 | 2.88 (1.69) | 2.90 (1.69) | 2.81 (1.68) | 0.542 | 2.60 (1.70) | 2.58  (1.63) | 2.68  (1.91) | 0.533 |
|  |  |  |  |  |  |  |  |  |  |  |  |  |
| ***Harsh Discipline at school:*** teacher threatened or hit child with a cane | 15.4% | 15.0% | 16.7% | 0.706 | 8.1% | 7.6% | 9.9% | 0.446 | 15.7% | 15.4% | 16.7% | 0.787 |

| **Supplementary Table S4**  *Descriptive statistics of harsh discipline at home and school by waves, stratified by child school type (public vs private) at respective wave (N = 663)* | | | | | | | | | | | | |
| --- | --- | --- | --- | --- | --- | --- | --- | --- | --- | --- | --- | --- |
|  | **2016** | | | | **2017** | | | | **2018** | | | |
|  | ***Average*** | ***Public*** | ***Private*** | ***p*** | ***Average*** | ***Public*** | ***Private*** | ***p*** | ***Average*** | ***Public*** | ***Private*** | ***p*** |
| ***Physical punishment*** |  |  |  |  |  |  |  |  |  |  |  |  |
| Adult shook child | 27.5% | 33.5% | 27.4% | 0.133 | 22.0% | 21.1% | 24.9% | 0.305 | 24.9% | 27.1% | 22.9% | 0.267 |
| Adult spanked, hit, or slapped child in the bottom with bare hands | 48.6% | 52.3% | 53.9% | 0.759 | 50.4% | 53.8% | 49.8% | 0.372 | 38.0% | 38.0% | 39.1% | 0.842 |
| Adult hit child in the bottom or elsewhere in the body with hard objects | 44.6% | 53.1% | 44.6% | **0.049** | 44.6% | 43.9% | 48.0% | 0.355 | 29.6% | 31.5% | 28.9% | 0.526 |
| Adult hit or slapped child on the face, head, or ears | 10.1% | 13.8% | 8.4% | **0.049** | 7.8% | 5.5% | 11.4% | **0.012** | 6.6% | 8.6% | 5.4% | 0.151 |
| Adult hit or slapped child on the hand, arm or leg | 49.9% | 56.9% | 53.3% | 0.428 | 44.0% | 47.1% | 44.0% | 0.483 | 42.5% | 44.5% | 41.4% | 0.467 |
| Adult beat child repeatedly as hard as they could | 9.0% | 11.2% | 8.7% | 0.399 | 6.0% | 5.5% | 7.7% | 0.347 | 6.9% | 7.9% | 6.5% | 0.607 |
| *Total physical punishment items [M (SD)]* | 2.06 (1.46) | 2.21  (1.51) | 1.96  (1.41) | **0.043** | 1.81 (1.37) | 1.8  (1.3) | 1.9  (1.5) | 0.434 | 1.50 (1.35) | 1.58  (1.41) | 1.44  (1.31) | 0.214 |
|  |  |  |  |  |  |  |  |  |  |  |  |  |
| ***Psychological aggression*** |  |  |  |  |  |  |  |  |  |  |  |  |
| Adult shouted, yelled at or screamed at child | 76.2% | 78.5% | 83.1% | 0.182 | 78.7% | 85.0% | 74.7% | **0.002** | 78.4% | 76.4% | 80.7% | 0.210 |
| Adult called child dumb, lazy, or similar | 23.7% | 27.7% | 23.2% | 0.247 | 25.5% | 25.1% | 28.6% | 0.387 | 30.8% | 31.5% | 30.9% | 0.931 |
| *Total psychological aggression items [M (SD)]* | 1.06 (0.62) | 1.06  (0.66) | 1.06  (0.59) | 0.974 | 1.07 (0.62) | 1.10  (0.58) | 1.03  (0.67) | 0.182 | 1.10 (0.66) | 1.08  (0.68) | 1.12  (0.66) | 0.478 |
| *Total harsh discipline (physical & psychological items combined) [M (SD)]* | 3.17  (1.77) | 3.27  (1.88) | 3.03  (1.67) | 0.103 | 2.88 (1.69) | 2.87  (1.56) | 2.89  (1.83) | 0.885 | 2.60 (1.70) | 2.65  (1.75) | 2.56  (1.66) | 0.476 |
|  |  |  |  |  |  |  |  |  |  |  |  |  |
| ***Harsh Discipline at school:*** teacher threatened or hit child with a cane | 15.4% | 21.2% | 12.3% | **0.006** | 8.1% | 9.2% | 7.7% | 0.588 | 15.7% | 22.3% | 9.3% | **<.001** |

**Supplementary Table S5**

*Exploratory analysis of harsh discipline at home and school predicting each of children’s developmental domains (N = 663)*

|  | **Literacy** | **Numeracy** | **Cognitive Flexibility** | **Inhibitory Control** | **Behavioral Regulation** |
| --- | --- | --- | --- | --- | --- |
|  | **𝛽 (SE)** | **𝛽 (SE)** | **𝛽 (SE)** | **𝛽 (SE)** | **𝛽 (SE)** |
| **Model 1** |  |  |  |  |  |
| Cumulative harsh discipline at home | -0.033 (0.025) | -0.056 (0.023)* | 0.005 (0.030) | -0.028 (0.032) | -0.081 (0.032)* |
| Intercept | -1.434 (0.284)*** | -1.160 (0.264)*** | -0.719 (0.340)* | -0.748 (0.369)* | -0.128 (0.313) |
| R^2^ | 0.369 | 0.454 | 0.028 | 0.024 | 0.099 |
|  |  |  |  |  |  |
| **Model 2** |  |  |  |  |  |
| Cumulative harsh discipline at school | -0.004 (0.045) | -0.063 (0.041) | -0.070 (0.055) | 0.069 (0.051) | -0.082 (0.058) |
| Intercept | -1.570 (0.260)*** | -1.365 (0.244)*** | -0.673 (0.331)* | -0.889 (0.365)* | -0.175 (0.317) |
| R^2^ | 0.367 | 0.451 | 0.030 | 0.025 | 0.092 |
|  |  |  |  |  |  |
| **Model 3** |  |  |  |  |  |
| Cumulative harsh discipline at home | -0.034 (0.025) | -0.058 (0.023)* | 0.003 (0.030) | -0.026 (0.032) | -0.083 (0.031)** |
| Cumulative harsh discipline at school | -0.007 (0.046) | -0.068 (0.041)† | -0.070 (0.055) | 0.066 (0.051) | -0.089 (0.058) |
| Intercept | -1.431 (0.287) | -1.127 (0.267)*** | -0.685 (0.343)* | -0.780 (0.371)* | 0.171 (0.316) |
| R^2^ | 0.368 | 0.455 | 0.029 | 0.024 | 0.102 |
|  |  |  |  |  |  |
| **Model 4** |  |  |  |  |  |
| Cumulative harsh discipline at home | -0.044 (0.029) | -0.073 (0.027)** | -0.032 (0.034) | -0.029 (0.037) | -0.090 (0.037)* |
| Cumulative harsh discipline at school | -0.089 (0.102) | -0.193 (0.096)* | -0.361 (0.153)* | 0.040 (0.140) | -0.152 (0.139) |
| Cumulative harsh discipline at home x Cumulative harsh discipline at school | 0.029 (0.035) | 0.044 (0.032) | 0.103 (0.046)* | 0.009 (0.048) | 0.022 (0.047) |
| Intercept | -1.400 (0.289) | -1.080 (0.270)*** | -0.576 (0.346) | -0.770 (0.377)* | 0.194 (0.332) |
| R^2^ | 0.368 | 0.456 | 0.034 | 0.023 | 0.101 |

*Note.* SE = standard error. All coefficients are standardized (𝛽). Clustered standard errors are shown in parentheses. Analyses controlled for full covariates (coefficients not shown). †*p* <. 10; **p* < 0.05; ***p* < 0.01; ****p* < 0.001.

**Supplementary Figure S1**

*Moderating effects of cumulative harsh discipline at school on the relations between cumulative harsh discipline at home and children’s cognitive flexibility skills*

*Note.* SE = standard error; SD = standard deviation. The solid line indicates high levels (+1 SD) of cumulative harsh discipline at school; the dashed line indicates low levels (-1 SD) of cumulative harsh discipline at school. Under conditions of high levels of cumulative harsh discipline at school, there were no differences in cognitive flexibility by exposure to cumulative harsh discipline at home (𝛽 = 0.047, *SE* = 0.045, *p* = .294). For children experiencing lower levels of cumulative harsh discipline at school, higher levels of cumulative harsh discipline at home were significantly associated with lower cognitive flexibility (𝛽 = -0.084, *SE* = 0.042, *p* = .044).

**Appendix**

**Discipline Module, adapted from the Parental Discipline Practices questionnaire in the *Multiple Indicator Cluster Survey* by UNICEF**

|  | **I. DISCIPLINE** | |
| --- | --- | --- |
| I01. | I will now ask you some questions about how you or any adult in your household discipline your child. Adults use certain ways to teach children the right behavior or to address a behavior problem. I will read various methods that are used. | |
| a. | Please tell me if you or someone else in your household took away privileges, forbade something [CHILD] liked (e.g., watching TV, playing with friends) or did not allow [CHILD] to leave the house in the past month? | 1. [ ] Yes  2. [ ] No  -888. [ ] Don't know  -999. [ ] Refuse to answer |
| b. | Please tell me if you or someone else in your household explained why [CHILD]’s behavior was wrong in the past month? | 1. [ ] Yes  2. [ ] No  -888. [ ] Don't know  -999. [ ] Refuse to answer |
| c. | Please tell me if you or someone else in your household shook [CHILD] in the past month? | 1. [ ] Yes  2. [ ] No  -888. [ ] Don't know  -999. [ ] Refuse to answer |
| d. | Please tell me if you or someone else in your household shouted, yelled at or screamed at [CHILD] in the past month? | 1. [ ] Yes  2. [ ] No  -888. [ ] Don't know  -999. [ ] Refuse to answer |
| e. | Please tell me if you or someone else in your household gave [CHILD] something else to do in the past month? | 1. [ ] Yes  2. [ ] No  -888. [ ] Don't know  -999. [ ] Refuse to answer |
| f. | Please tell me if you or someone else in your household spanked, hit or slapped [CHILD] on the bottom with bare hand in the past month? | 1. [ ] Yes  2. [ ] No  -888. [ ] Don't know  -999. [ ] Refuse to answer |
| g. | Please tell me if you or someone else in your household hit [CHILD] on the bottom or elsewhere on the body with something like a belt, hairbrush, stick or other hard object in the past month? | 1. [ ] Yes  2. [ ] No  -888. [ ] Don't know  -999. [ ] Refuse to answer |
| h. | Please tell me if you or someone else in your household called [CHILD] dumb, lazy, or another name like that in the past month? | 1. [ ] Yes  2. [ ] No  -888. [ ] Don't know  -999. [ ] Refuse to answer |
| i. | Please tell me if you or someone else in your household hit or slapped [CHILD] on the face, head or ears in the past month? | 1. [ ] Yes  2. [ ] No  -888. [ ] Don't know  -999. [ ] Refuse to answer |
| j. | Please tell me if you or someone else in your household hit or slapped [CHILD] on the hand, arm, or leg in the past month? | 1. [ ] Yes  2. [ ] No  -888. [ ] Don't know  -999. [ ] Refuse to answer |
| k. | Please tell me if you or someone else in your household beat [CHILD] up, that is hit him/her over and over as hard as one could in the past month? | 1. [ ] Yes  2. [ ] No  -888. [ ] Don't know  -999. [ ] Refuse to answer |
| I02. | Do you believe that in order to bring up, raise, or educate a child properly, the child needs to be physically punished? | 1. [ ] Yes  2. [ ] No  -888. [ ] Don't know  -999. [ ] Refuse to answer |
